# Molecular switches and cages

**DOI:** 10.3762/bjoc.8.97

**Published:** 2012-06-13

**Authors:** Dirk Trauner

**Affiliations:** 1 Department Chemie und Biochemie, Ludwig-Maximilians-Universität München, Butenandtstr. 5–13, 81377 München, Germany

**Keywords:** molecular switches

Molecular switches can toggle between two (or more) stable states encoding different physical features. They allow complex systems to respond to changes in their environment defined by light intensity, pH, temperature or voltage. As such, molecular switches play a key role in biology and information technology and have become important components of advanced materials.

Chemists have developed a large number of synthetic switches, complementing the many types found in nature. Given their prominence in biology, it is not surprisingly that photoswitches, which are actuated by ultraviolet, visible or infrared light, have been an especially productive field of study. Despite their long history, new types of synthetic photoswitches continue to emerge and “old friends”, such as azobenzenes are constantly improved and optimized. This is not only true with respect to their photophysical properties but also with regard to their synthetic accessibility. Indeed, as molecular switches are applied in ever-increasing quantities, the efficiency of their syntheses becomes a primary concern, requiring the development of new synthetic methods and strategies. The corresponding results, however, are often buried in the Supporting Information of papers that focus on functional aspects, which does not reflect the relative effort that goes into the design and synthesis of these molecules.

Caged compounds are a subclass of (photo)switches that have special functional features. As opposed to “true” switches, they can only be turned ON and the abatement of their activity depends on secondary processes (such as diffusion from the active zone or, more importantly, re-uptake systems). In terms of their biological application, especially in neurobiology, they are much further developed than reversibly switchable molecules. For instance, two-photon activation, which allows for very precise localization, is fairly well established with caged compounds but in its infancy in the case of reversible photoswitches.

The present Thematic Series of the Beilstein Journal of Organic Chemistry addresses the crucial role that chemistry, and particularly organic chemistry, can play in tuning the functional features of molecular switches and cages. These features include their absorption spectra, conductivity, geometry and bistability, as well as their polarity, solubility, efficacy, or catalytic activity. Photoswitches are covered extensively, ranging from diarylethenes (Pu, Wagenknecht) to dihydroazulenes/vinylheptafulvenes (Nielsen) and azobenzenes (Rück-Braun, Hoppmann). Two reviews discuss *cis*-azobenzenes with rapid thermal isomerization kinetics (Velasco), and highlight the azobenzene moiety as one of the smallest light-driven molecular motors conceivable (Merino). Issues of bistability are also addressed in an account on shape-persistent, optically active macrocycles (Pasini). Molecular switches that respond to changes in pH are covered as well (Haberhauer and Aprahamian), reflecting their importance in biology and in materials science. Finally, switching is highlighted as a synthetic strategy for building advanced materials that are useful in photovoltaics (Matile).

Chemists, by nature, like to control things and probably actuate macroscopic switches more often than the average person. We hope that this collection of papers will motivate some of our colleagues to also consider nanoscopic versions of switches (and cages). For those of us who deal with these molecules on a daily basis, this Thematic Series will hopefully provide further inspiration and an impetus to continue work in this fascinating field of research.

Dirk Trauner

Munich, June 2012

